# A 3-year observation of testosterone deficiency in Chinese patients with chronic heart failure

**DOI:** 10.18632/oncotarget.19816

**Published:** 2017-08-02

**Authors:** Ying Han, Weiju Sun, Guizhi Sun, Xiaolu Hou, Zhaowei Gong, Jing Xu, Xiuping Bai, Lu Fu

**Affiliations:** ^1^ Cardiovascular Department, The Fourth Affiliated Hospital of Harbin Medical University, Harbin 150001, China; ^2^ Cardiovascular Department, The First Affiliated Hospital of Harbin Medical University, Harbin 150001, China

**Keywords:** testosterone deficiency, chronic heart failure, mortality, readmission

## Abstract

Testosterone deficiency is present in a certain proportion men with chronic heart failure (CHF). Low testosterone levels in American and European patients with CHF lead to the high mortality and readmission rates. Interestingly, this relationship has not been studied in Chinese patients. To this end, 167 Chinese men with CHF underwent clinical and laboratory evaluations associated with determinations of testosterone levels. Total testosterone (TT) levels and sex hormone-binding globulin were measured by chemiluminescence or immunoassays assays and free testosterone (FT) levels were calculated, Based upon results from these assays, patients were divided into either a low testosterone (LT; *n* = 93) or normal testosterone (NT; *n* = 74) group. Subsequently, records from each patient were reviewed over a follow-up duration of at least 3 years. Patients in the LT group experienced worse cardiac function and a higher prevalence of etiology (ischemic vs. no ischemic) and comorbidity (both *P* < 0.05). In addition, readmission rates of patients in the LT group were higher than that of patients in the NT group (3.32 ± 1.66 VS 1.57 ± 0.89). Overall, deficiencies in FT levels were accompanied with increased mortalities (HR = 6.301, 95% CI 3.187–12.459, *P* < .0001).

## INTRODUCTION

Chronic heart failure (CHF) is a major health problem throughout the world as the final outcome of almost all cardiovascular diseases [1–11]. It is associated with a high rate of hospitalization and a devastating prognosis, despite the availability of modern combinational therapies. The mortality rate of CHF is higher than that of most cancers, and more than 50% of CHF patients die within 5 years of diagnosis [12]. Different clinical feature were observed in men and women with CHF, such as symptoms, response to therapies, and mortality [13–15]. Statistical results show that more number of women than men suffer from CHF. Whereas, women with CHF can benefit much more from medication and have a higher age-adjusted survival rate than men. Therefore, it is reasonable to present the hypothesis that sex hormones may play a role in CHF.

Previous studies [16–18] have shown that a testosterone deficiency existed in men with CHF, and serum testosterone correlated inversely with ventricular ejection fraction, hemodynamics and exercise capacity. Moreover, deficiencies in anabolic hormones (testosterone, dehydroepiandrosterone sulfate, and insulin-like growth factor-1) can serve as predictors for poor prognosis [19]. Further evidence for this has emerged from several trials [20–22] demonstrating that testosterone replacement therapy is associated with significant increases in exercise tolerance, improvement of symptoms and amendments in the quality-of-life. To our knowledge, there are few data [19, 23–25] on the relationship between testosterone levels and 3-year outcomes (mortality and readmission rates) in men with CHF and yielded conflicting results. The aim of the our study was to evaluate the prospective effect of testosterone deficiency on the 3-year mortality and readmission rates in male patients hospitalized with CHF.

## RESULTS

### Hormone levels and serum parameters

Baseline characteristics of the 167 Chinese patients with CHF are summarized in Table 1. Patients in LT group were characterized by older age, higher systolic blood pressure, advanced New York Heart Association (NYHA) class, lower left ventricular ejection fraction (LVEF), lager left ventricular end diastolic diameter (LVEDD), decreased hemoglobin, decreased estimated glomerular filtration rate (GFR), increased serum B-type natriuretic peptide (BNP) level, and higher prevalence of etiology(ischemic vs. no ischemic) and comorbidities (atrial fibrillation, hypertension and diabetes). All the patients were treated with standard drug regimens, including diuretics, angiotensin-converting-enzyme (ACE) inhibitors or angiotensin receptor blocker (ARB), β-Blockers, digoxin and Spironolactone.

**Table 1:** Physical, clinical, and hormonal characteristics of patients with heart failure at the first admission

Variables	Low Testosterone (*n* = 93)	Normal Testosterone (*n* = 74)	statistics	*P* value
Age, y	69(60–75)	60(50–71)	3.43	0.0006
NYHA class, n%			5.78	<.0001
II	5(5.38)	35(47.30)		
III	42(45.16)	24(32.43)		
IV	46(49.46)	15(20.27)		
LVEF, %	39.0(32.0–42.0)	44.5(39.0–46.0)	4.75	<.0001
LVEDD, mm	64(60–68)	60(58–65)	3.02	0.0025
TT, ng/dL	239.14(197.30–281.01)	401.19(332.14–502.63)	11.08	<.0001
FT, pmol/L	110.36(81.17–133.23)	175.15(146.68–201.78)	6.73	<.0001
SBP, mmHg	158(138–169)	135(118–156)	3.60	0.0003
Ischemic, n%	78(83.87)	50(67.57)	6.12	0.0134
Comorbidities, n%				
Atrial fibrillation	37(39.78)	12(16.22)	11.04	0.0009
Hypertension	73(78.49)	40(54.05)	11.25	0.0008
Diabetes	48(51.61)	14(18.92)	18.87	<.0001
Medication, n%				
ACEI/ARB	74(79.57)	71(95.95)	9.66	0.0019
β-blocker	83(59.25)	65(87.84)	0.08	0.7757
Digoxin	43(46.24)	24(32.43)	3.27	0.0706
Diuretic	90(96.77)	43(58.11)	38.00	<.0001
Laboratory variables				
GFR,mL·min^–1^·1.73 m^2^	65.8(54.8–77.2)	84.9(74.9–90.8)	6.36	<.0001
BNP, pg/mL	2105.0(1368.0–2968.0)	1134.5(945.0–1697.0)	6.22	<.0001
Hemoglobin, g/L	160(146–169)	151(138–162)	2.68	0.0073

Results are presented as median (with upper and lower quartiles) or % where appropriate. NYHA, New York Heart Association; LVEF: Left ventricular ejection fraction; LVEDD: left ventricular end diastolic diameter; TT: Total testosterone; FT: Free testosterone; ACE-I/ARB: Angiotensin-converting enzyme inhibitor/angiotensin receptor blocker; GFR: Glomerular filtration rate; BNP: B-type natriuretic peptide.

### Clinical outcomes

**Figure 1:**
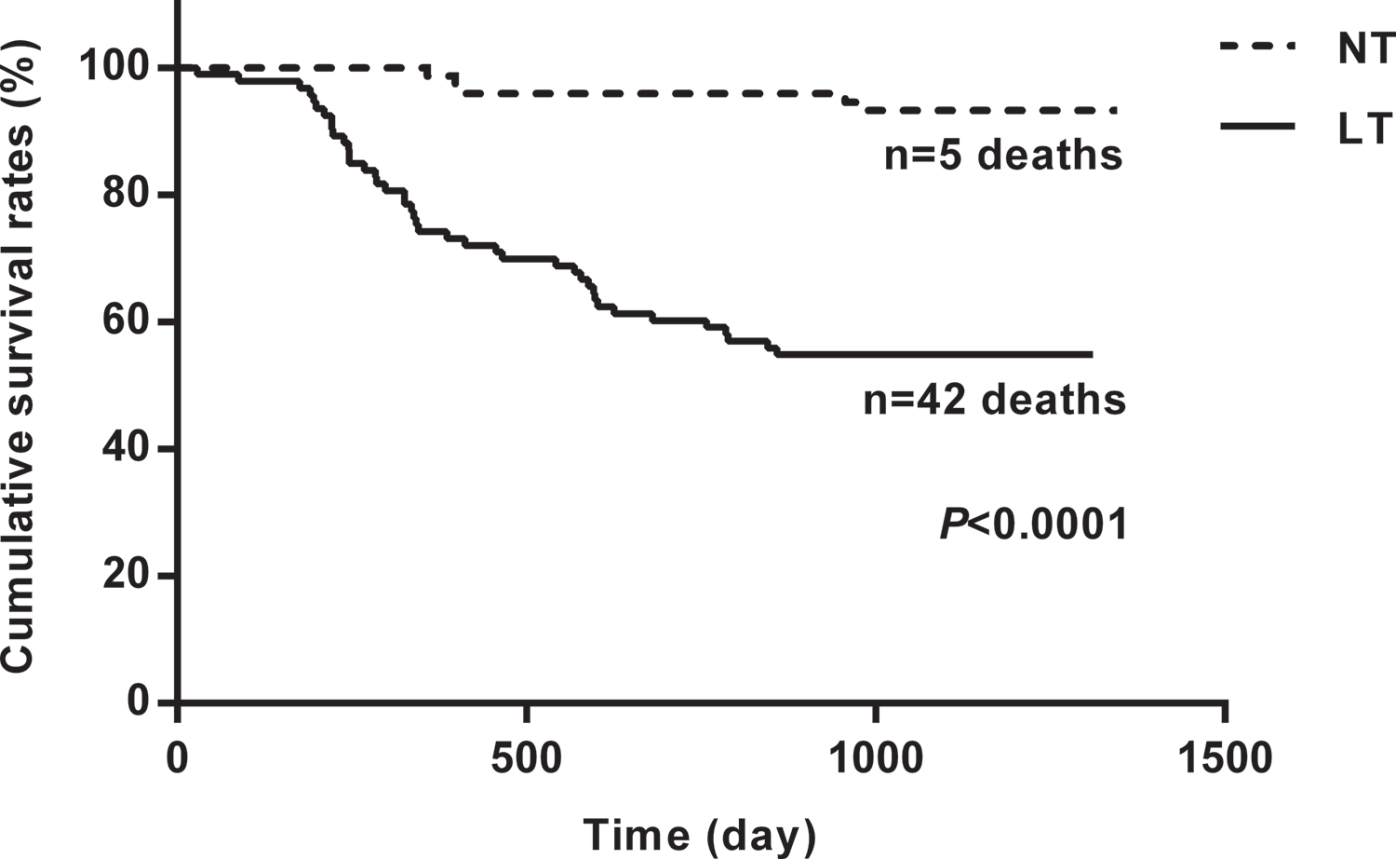
Kaplan-Meier curves for cumulative survival rates of patients in the two groups The low testosterone group showed significantly higher mortality rates as compared with that of the normal testosterone group (*P* < .0001).

**Figure 2:**
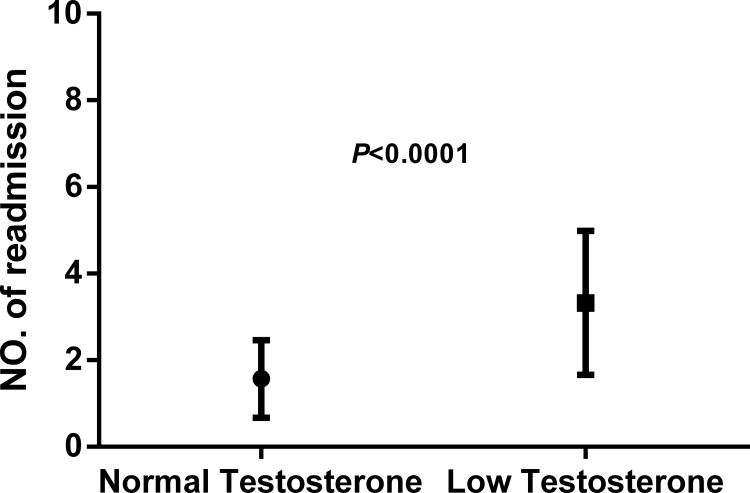
Readmission rates as determined over the 3-year follow-up period in patients with heart failure The low testosterone group showed significantly higher readmission rates as compared with that of the normal testosterone group (*P* < .0001).

**Figure 3:**
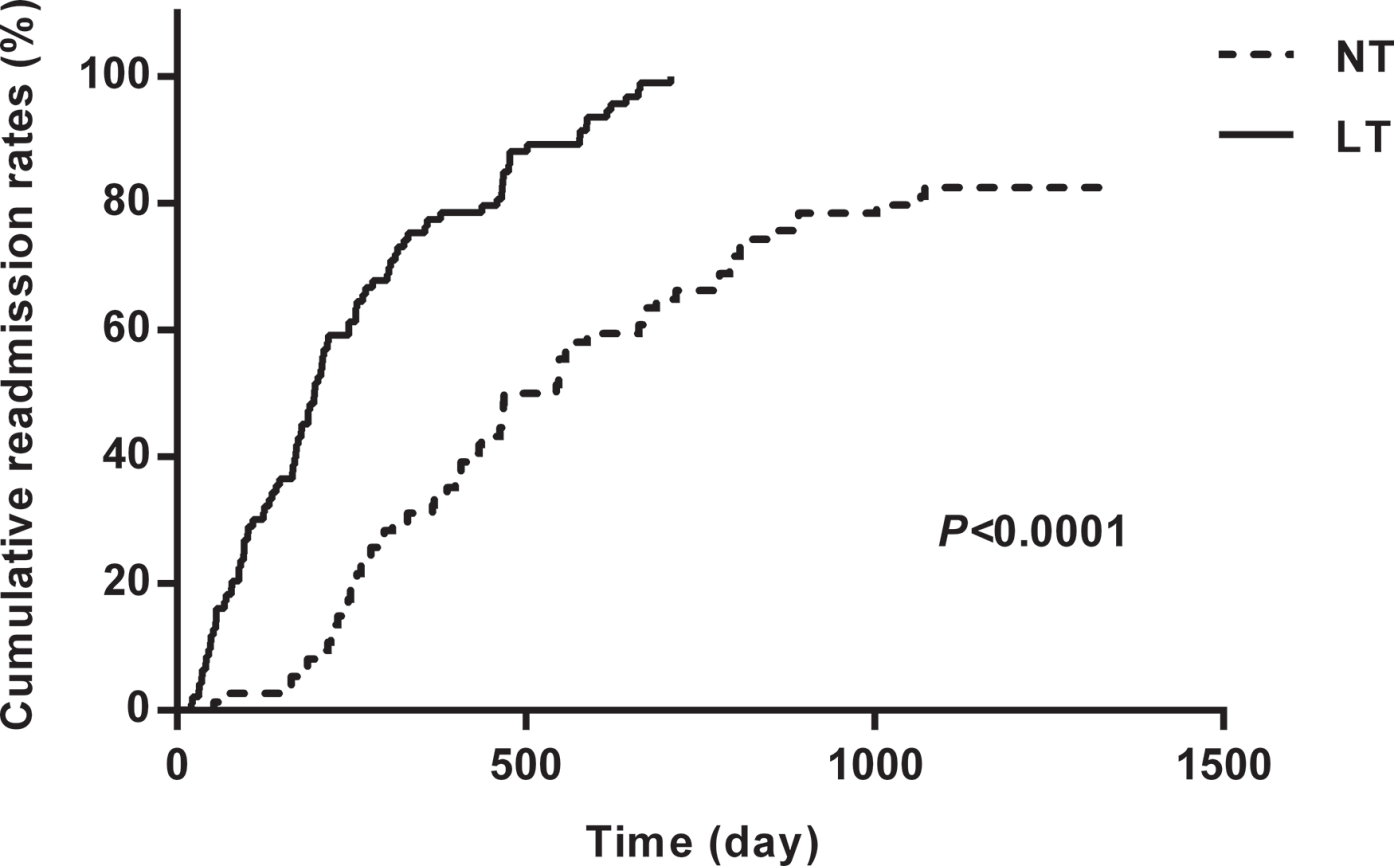
Kaplan-Meier curves for cumulative readmission rates for patients in the two groups The low testosterone group showed significantly higher readmission rates as compared with that of the normal testosterone group ($\chi^{2}$ = 56.57, *P* < .0001).

At the end of the 3-year follow-up period, there were 47 deaths (28% of all patients enrolled) with all deaths being cardiovascular. The LT group experienced a significantly higher mortality rate (42/93) as compared to the NT group (5/74) at this 3-year follow-up period (Table 2). This mortality rate was significantly greater in the LT versus NT group ($\chi^{2}$ = 30.60, *P* < .0001) as shown in the Kaplan-Meier curves for cumulative survival rates of patients in the two groups (Figure 1). Of surviving patients, 86% experienced at least one unplanned admission resulting from a worsening of heart failure. Readmission rates were significantly greater in the LT (3.32 ± 1.66) as compared to the NT (1.57 ± 0.89) group (Table 2, Figure 2). Figure 3 shows the Kaplan-Meier curves for cumulative readmission rates for patients in the two groups. The LT group had a significantly higher readmission rates compared to the NT group ($\chi^{2}$ = 56.57, *P* < .0001).

**Table 2:** Clinical outcomes

Variables	Low Testosterone (*n* = 93)	Normal Testosterone (*n* = 74)	statistics	*P* value
Age,y	66.75 ± 11.63	60.09 ± 12.66	3.53	0.0005
follow-up time,d	818.2 ± 406.6 1089(345–1154)	1146.0 ± 178.7 1147(1111–1248)	5.06	<.0001
Readmission times	3.32 ± 1.66 3(2–4)	1.57 ± 0.89 1(1–2)	8.72	<.0001
death	42(45.16)	5(6.76)	30.06	<.0001

### Prognostic factors

Prognostic results of the univariate Cox proportional hazard analysis to predict cardiac death are shown in Table 3. Reduced levels of TT and FT in serum were identified as available predictors of mortality in men with CHF (TT, HR = 0.885, 95% CI 0.829–0.945, *P* = .0003; FT, HR = 13.360, 95% CI 7.149–24.967, *P* < .0001). In multivariable models, we included nearly all the variables that were involved in the univariate analyses. The final model included 3 significant independent predictors consisting of LVEF (HR = 1.075, 95% CI 1.026–1.127, *P* = 0.0003), serum FT (HR = 6.301, 95% CI 3.187–12.459, *P* < .0001), and GFR (HR = 0.967, 95% CI 0.937–0.998, *P* = 0.0348) (Table 4).

**Table 3:** Single-predictor models of the Cox proportional hazard analysis for mortality

Variables	B	SE	$\chi^{2}$	HR	95% CI	*P* value
Age,y	0.0045	0.0118	0.1445	1.004	0.982–1.028	0.7038
NYHA class	2.5400	0.4181	36.9092	12.679	5.588–28.772	< .0001
LVEF, %	–0.1703	0.0185	84.6558	0.843	0.813–0.875	< .0001
LVEDD, mm	0.1183	0.0158	55.7897	1.126	1.091–1.161	< .0001
TT, ng/dL	2.1715	0.4737	21.0173	8.771	3.466–22.194	< .0001
FT, pmol/L	2.5923	0.3190	66.0283	13.360	7.149–24.967	< .0001
SBP, mmHg	–0.0005	0.0058	0.0085	0.999	0.988–1.011	0.9267
Ischemic	–0.0482	0.3446	0.0196	0.953	0.485–1.872	0.8887
Atrial fibrillation	0.4733	0.3003	2.4851	1.605	0.891–2.892	0.1149
Hypertension	–0.1333	0.3079	0.1874	0.875	0.479–1.600	0.6651
Diabetes	1.1237	0.2978	14.2425	3.076	1.716–5.514	0.0002
ACEI/ARB	–1.3879	0.3206	18.7353	0.250	0.133–0.468	< .0001
β-blocker	0.7487	0.5968	1.5741	2.114	0.656–6.810	0.2096
Digoxin	1.0142	0.3003	11.4071	2.757	1.531–4.967	0.0007
Diuretic	2.0411	0.7226	7.9774	7.699	1.868–31.734	0.0047
GFR,mL·min^–1^·1.73 m^2^	–0.05911	0.0078	57.3415	0.943	0.928–0.957	< .0001
BNP, pg/mL	0.0012	0.0001	102.6056	1.001	1.001–1.001	< .0001
Hemoglobin, g/L	0.00134	0.0062	0.0470	0.999	0.987–1.011	0.8284

NYHA, New York Heart Association; LVEF: Left ventricular ejection fraction; LVEDD: left ventricular end diastolic diameter; TT: Total testosterone; FT: Free testosterone; ACE-I/ARB: Angiotensin-converting enzyme inhibitor/angiotensin receptor blocker; GFR: Glomerular filtration rate; BNP: B-type natriuretic peptide.

**Table 4:** Multivariable models of Cox proportional hazard analyses for mortality

Variables	B	SE	$\chi^{2}$	HR	95% CI	*P* value
LVEF	–0.1222	0.0336	13.2309	0.885	0.829–0.945	0.0003
FT	1.8407	0.3478	28.0117	6.301	3.187–12.459	< .0001
GFR	–0.0339	0.0161	4.4534	0.967	0.937–0.998	0.0348

The meaningful variables in the multivariable models were listed. LVEF: Left ventricular ejection fraction; TT: Total testosterone; FT: Free testosterone; GFR: Glomerular filtration rate.

## DISCUSSION

The salient results in this study of male Chinese patients with CHF include two major findings. First, CHF patients with decreased TT and FT concentrations experienced worse heart function compared with that in normal individuals. Second, a deficiency in testosterone was an independent risk predictor for mortality and readmission rates in men hospitalized with CHF.

Several published studies have suggested that patients with CHF had low androgen levels. In the present study, we determined serum levels of TT, which is the major component of androgens, and as well as FT, which reflects the most active fraction of circulating gonadal androgens and is directly related to biological effects on target tissues. Here we report that patients in LT group had worse cardiac function and higher prevalence of etiology (ischemicvs. no ischemic) and comorbidities (atrial fibrillation, hypertension and diabetes).

An important component of our current findings is that this androgen deficiency in men with CHF is not only a simple surrogate of disease severity, but also an independent risk factor of prognosis. Data from previous studies have suggested that testosterone insufficiency was associated with an increasing risk of death due to all cardiovascular related diseases in men [26–28]. Jankowska et al. [19] investigated the prevalence and prognostic consequences of deficiencies in circulating TT, FT, dehydroepiandrosterone sulfate (DHEAS), and insulin-like growth factor-1 in men with CHF. Their results showed that men with CHF and normal levels of all anabolic hormones experienced the best 3-year survival rate (83%) as compared to those with deficiencies in 1 (74% survival rate), 2 (55% survival rate), or all 3 (27% survival rate) anabolic endocrine indices. However, another two studies [24, 25] found that after adjustment of variables that were associated with both the exposure and the outcome, neither TT nor FT, DHEAS or sex hormone-binding globulin (SHBG) represent independent predictors of mortality risk. More recently, results from Marcelo et al. [23] in their multivariable analyses of 110 male patients with CHF, also provide support for the concept that LT is an independent risk factor for hospital readmission within 90 days and increased mortality in patients with HF. In our present study, as well as in that reported by Jankowska et al. and Marcelo et al., reduced levels of TT and FT were related to an increased 3-year mortality rate (all cardiovascular deaths) in men with CHF. Taken together, these results suggest that androgen deficiency is likely to be one of the reasons leading to aggravation and deterioration in the progression of heart failure. However, others [5, 6] have found that after adjustment of variables that were associated with both the exposure and the outcome, neither TT nor FT, DHEAS or sex hormone-binding globulin (SHBG) represent independent predictors of mortality risk. Such disparate findings can in part, be explained by differences in the samples of CHF patients enrolled and measurements of TT levels. For example, the sample studied by Guder et al. [5, 6] included a large proportion of CHF patients with preserved LVEF, unlike those in the other studies. The measurement of TT levels in the study by Wu et al. [5, 6] used a electrochemiluminescence assay, which may have resulted in an overestimation of TT levels. In addition, differences in parameters measured among these studies can contribute to contrasting conclusions. In the study by Jankowska et al. measures of additional hormones, such as DHEAS and insulin-like growth factor-1 were included in their analysis, while the study by Guder et al. included additional prognostic measures, such as medication and inflammatory markers, that were not included in Jankowska et al. There are more additional prognostic variables including the Comorbidities.

It is well known that testosterone exerts effects on contractile function of myocardium, vasodilation, insulin resistance, inflammation, coagulation, obesity, endothelial function and alteration in skeletal muscle [29–35]. In addition, testosterone concentration in serum reflects one aspect of anabolic insufficiency. Anabolic/catabolic imbalances not only can result in the activation of catabolism but the inhibition of anabolism, as well as the activation of the neuroendocrine and cytokine systems, which play an important role in the pathological processes of CHF. Such imbalances are related to the symptoms, signs and cardiac cachexia [36]. Therefore, it seems likely that the prognosis and high readmission rates associated with testosterone deficiency are the consequences of various and complex mechanisms that await further research [37, 38].

## MATERIALS AND METHODS

### Subjects

The study was performed between September 2009 and October 2011 in male Chinese patients hospitalized with CHF in Harbin. Male patients (*N* = 167), aged 36 to 87 years with NYHA functional class II, III or IV (40/66/61), participated in the study. The criteria for study enrollment were as follows: > 6 month symptomatic CHF; left ventricular ejection fraction (LVEF) ≤ 45% as assessed by echocardiogramphy; clinical stability and standard medications for at least 1 month preceding the study; no testosterone replacement or drugs that could affect testosterone levels, such as finasteride, opiates, glucocorticoids and anticonvulsants. In addition, enrollment required that the patients have an absence of heavy alcohol consumption, nephrotic syndrome, liver cirrhosis or cancer history.

The study protocol was approved by the ethics committees of the participating hospitals and all patients provided written informed consent.

### Hormonal assays and laboratory measurements

Venous blood samples were collected in the morning between 06:00 and 07:00 following an overnight fast and after a supine rest of at least 15 minutes on the second day after hospital admission. Serum concentrations of total testosterone (TT) were measured by chemiluminescence (Siemens Corp., USA). The inter and intra assay variability coefficients for TT were 2.5% and 5.8%, respectively. Serum levels of SHBG were measured with ELISA (BL, Hamburg, Germany), and the inter and intra-assay variability coefficients were 5.2% and 3.0%, respectively. Serum levels of free testosterone (FT) were then calculated using the validated equation of Vermeulen et al. [39]. Testosterone deficiency, referred to as hypogonadism, was defined as a total testosterone < 300 ng/dL and free testosterone < 131 pmol/L [40, 41].

All CHF patients underwent clinical and laboratory evaluations, including physical examination, electrocardiogram, echocardiography, chest X-ray, full blood count, serum electrolytes and, if necessary, coronary angiography. Measurements, such as BNP and hemoglobin, were performed by the hospital biochemistry laboratory. Estimated GFR was calculated using the formula from the Modification of Diet in the Renal Disease Study Group.

### Clinical follow-up

Patients were reviewed regularly, with a follow-up duration extending for at least 3 years. The study investigators were provided with information regarding mortality and readmission rates (as of October 30, 2014) directly from patients and their relatives by periodic phone calls, or from the hospital system. No patient was lost to follow-up. The two endpoints included: (1) worsening of heart failure as indicated by readmission and/or (2) cardiac death, defined as death from worsening heart failure or sudden cardiac death.

### Statistical analysis

Most variables showed a skewed distribution and were therefore expressed as medians with lower and upper quartiles. Comparisons between the two groups were made using the Mann–Whitney U testor the χ2 test, where appropriate. The association of total and free testosterone levels with all cause of mortality was determined by Cox proportional hazards regression. The following variables were included in the Single-predictor models of the Cox proportional hazard analysis: age, etiology, NYHA class, LVEF, LVEDD, systolic blood pressure, comorbidities, medication, GFR, plasma BNP, hemoglobin and serum levels of TT and FT. During construction of the multivariable models, variables that may be significant predictors of survival according to the Single-predictor models and the clinic, were included in the analyses. These variables included: age, etiology, NYHA class, LVEF, LVEDD, systolic blood pressure, comorbidities, medication, GFR, BNP levels, and serum levels of TT and FT. In order to estimate the effect of serum levels of TT and FT on 3-year survival rates and readmission rates, Kaplan–Meier curves for cumulative survival and readmission rates were constructed for patients in the LT and NT groups. Comparisons of survival rates were tested with the Cox-Mantel log-rank test. Differences with a value of *P* < 0.05 were considered required for results to be statistically significant. The data analysis was conducted by an independent university-based statistician using SPSS 19.0 (Statistical Package for the Social Sciences 19.0, IBM SPSS company, USA).

## CONCLUSIONS

In this sample of Chinese men with CHF, testosterone deficiency served as a predictor for the high rates of 3-year mortality and readmission. Our findings suggested the potential for beneficial effects of testosterone in the treatment of this condition.

### Study limitation

There remain a number of limitations related to this study. First, the sample size was relatively small. Therefore, these data should be confirmed as based upon a larger sample of patients and preferably contain all NYHA functional classes especially class I. Second, patients enrolled in this study were limited to CHF patients with CHF who had experienced a significant cardiac event requiring hospitalization. Whether these findings would also be applicable to outpatients with stable clinical manifestations should be further investigation. Third, levels of FT and TT were measured at only one time point. It is not known whether FT and TT levels may fluctuate in CHF patients with CHF over the 3-year follow-up period. Nor is it known whether FT and TT levels decreased in NT patients over this 3-year period. Finally, our currents results do not provide any information regarding mechanisms by which testosterone deficiency predicts poor outcome and high readmission rate in men with CHF. Indeed, such mechanisms may involve a multi-factorial process and remains a subject for further investigation.
